# Characterization of Indigenous Lactic Acid Bacteria in Cow Milk of the Maltese Islands: A Geographical and Seasonal Assessment

**DOI:** 10.3390/microorganisms8060812

**Published:** 2020-05-28

**Authors:** Elisa Garroni, Agapi I. Doulgeraki, Foteini Pavli, David Spiteri, Vasilis P. Valdramidis

**Affiliations:** 1 Department of Food Sciences and Nutrition, Faculty of Health Sciences, University of Malta, MSD 2080 Msida, Malta; elisa_garroni101@hotmail.com (E.G.); photpavli@gmail.com (F.P.); dspit010@gmail.com (D.S.); 2 Institute of Technology of Agricultural Products, Hellenic Agricultural Organization-Demeter, S. Venizelou 1, 14123 Lycovrissi, Greece; 3 Centre of Molecular Medicine and Biobanking, University of Malta, MSD 2080 Msida, Malta

**Keywords:** milk, lactic acid bacteria, microbial diversity, geographical distribution, seasonality

## Abstract

A geographical and seasonal assessment of indigenous lactic acid bacteria (LAB) in Maltese cow milk was conducted in this study. To investigate this, milk was collected from different regions of Malta during winter and summer seasons. Total viable counts (TVC) and LAB population were enumerated. Afterwards, LAB were isolated and identified by molecular methods. According to the results, similar TVC were enumerated on winter and summer samples, while highest LAB population was detected on summer samples. LAB isolates were grouped in seven different clusters which were assigned to *Lactobacillus casei*, *Pediococcus* *pentosaceus*, *Lactobacillus* *plantarum*, *Weissella* *paramesenteroides*, *Lactobacillus* *rhamnosus*, *Lactococcus* *lactis,* and *Lactococcus* *garvieae*. In addition, *Enterococcus* and *Streptococcus* species were also isolated. Season seemed to affect the genus/species of LAB since *Lactobacillus* were mainly isolated from winter samples, while *Lactococcus* and *Enterococcus* species were the main genera identified in summer samples. Regarding the geographical distribution, the majority of the *Lactobacillus* spp. were isolated from the South-eastern region in both seasons. In conclusion, through this study the diversity of indigenous LAB in the Maltese cow milk was monitored for the first time and highlighted that the microbial communities are affected by seasonality and geographical distribution of the farms.

## 1. Introduction

The quality of raw milk is an important factor influencing the quality, safety, and economic performance of dairy products. Milk is a highly nutritious product, and thus it can serve as an optimum growth medium for a large variety of different microbes [1]. An increase in the shelf life of milk and other dairy products can serve as an initiative for local dairy industries to widen their distribution chain of products [2]. In the United States, the limits for the viable microorganisms in milk before pasteurization is 1 × 10^5^ CFU/mL for milk from an individual farmer, while for milk from multiple producers it is 3 × 10^5^ CFU//mL [3]. The type and quantity of microbes present both before and after pasteurization determine the overall quality of the product. The bacterial composition of raw milk can originate from the livestock that is used to produce the milk [4]. Most dairy animals are a reservoir of a large variety of microorganisms. Most of them are found in the digestive tract and are vital for the digestion of the food that the animal eats. However, an array of other microbes can be pathogenic and induce diseases [5]. Milk has the potential of serving as an optimum medium to support the growth of a wide range of microorganisms that are either harmful (e.g. *Brucella*, *Campylobacter*, *Cryptosporidium*, *Escherichia coli*, *Listeria,* and *Salmonella*) or beneficial (*Lactobacillus*, *Enterococcus*, and *Streptococcus*) [1]. LAB are especially important in the dairy industry, as they may possess at least one inherent functional property, and therefore can contribute to food safety and/or offer one or more sensorial, technological, nutritional, or health advantage. 

The aim of this study was to characterize the microbiota of raw Maltese cow milk for the first time and to assess the effect of season and region on microbial diversity. To our knowledge no information is available for the LAB diversity of raw milk from the country of Malta. The only available information about Maltese cow milk is its physicochemical characteristics [6,7]. To achieve our goal, the following objectives were set: (i) isolation for first time of native microbiota of raw Maltese milk with the main focus on lactic acid bacteria (ii) identification of the isolated autochthonous Maltese bacteria using molecular methods, (iii) comparison of the LAB isolated from raw Maltese milk in summer and winter and (iv) comparison of the LAB isolated from raw milk from five regions of Malta (Southern region, South-eastern region, Central region, Northern region and Gozo).

## 2. Materials and Methods 

### 2.1. Collection of Raw Milk Samples

A two factorial experiment was designed for the seasonal and geographical assessment of microbial communities of raw Maltese cow milk. Firstly, the Maltese Islands were divided into five regions, namely: Southern region, South-eastern region, Central region, Northern region, and Gozo. Raw milk samples were collected from all around Malta and Gozo and the region of the farms was noted. For the seasonal assessment, raw milk samples were collected from the local herdsmen in both winter (December, January, and February) of 2015 and summer (June, July, and August) of 2016. Samples were collected in a tightly sealed sterile plastic bottle using a sterile scoop directly from the herdsmen’s tanks as soon as the raw cow’s milk was delivered from the farm to the local dairy industry. 

### 2.2. Quantification and Isolation of Bacteria from Raw Cow’s Milk

The enumeration of bacteria from the raw milk samples was performed on the same day that the samples were collected. Raw milk aliquots of each herdsman were retrieved from the refrigerator (4 °C) and a serial dilution in ¼ strength Ringer‘s solution (Oxoid, Basingstoke, UK) was performed on each sample. The appropriate dilution was plated out on Tryptic Soya Agar (TSA) (Biolife, Milano, Italy) and Lactobacillus selective agar (LSA) (Himedia Laboratories, Mumbai, India) for the detection of Total Viable Counts (TVC) and LAB, respectively. The TVC and LAB population expressed on log cfu/ mL after incubation of TSA and LSA at 30 °C and 37 °C respectively for 48 h. After microbial enumeration, colonies from LSA medium were isolated; in order for the study to be close to a national survey, 10% of colonies grown on LSA were recovered and purified by successive subcultures on LSA. Purity of the cultures was also checked on Brain Heart Infusion agar (BHI) (Oxoid, UK), and pure isolates were stored in vials with TSB and glycerol (20% v/v) in a freezer at −80 °C, until further use. Prior use, each isolate was cultured in BHI broth at 37 °C for 24 h.

### 2.3. DNA Extraction and Strain Differentiation

DNA was extracted according to the protocol described by the Accuprep Genomic DNA extraction kit (Bioneer, Daejeon, Korea) and was stored in the −20 °C freezer until further use. For strain differentiation, all isolates were subjected to rep-PCR using primer GTG_5_ (GTGGTGGTGGTGGTG). PCR amplifications were conducted into a thermal cycler (Eppendorf AG 22331, Hamburg, Germany) in a final volume of 25 µL containing DNA TOP polymerase (Solis Biodyne, Estonia) (1.25 U), 1× blend mastermix buffer, dNTPs (0.2 mM from each), primer (2 µΜ), magnesium chloride (2 mM), DNA (2 µL) and ddH_2_O. PCR reactions consisted οf an initial denaturation step at 95 °C for 5 min, followed by 30 cycles of denaturation at 90 °C, 30 sec, primer annealing at 40 °C, 1 min and primer extension at 72 °C, 8 min, and concluded by a final extension step at 72 °C for 16 min. PCR products were separated in a 1.5% agarose gel 1× TAE buffer for 90 min at 100V. A 500 base pair ladder was chosen as marker. After visualization of bands under ultraviolet (UV) trans-illuminator, conversion, normalization, and further analysis were performed using the Sørensen–Dice coefficient and UPGMA (un-weighted pair group method with arithmetic mean) cluster analysis with Bionumerics software, version 6.1 (Applied Maths, Sint-Martens-Latem, Belgium). 

### 2.4. Species Identification 

Representative isolates with genetically different rep-PCR patterns i.e., 22 from the winter samples and 13 isolates from the summer samples were chosen for further analysis. The representative isolates were subjected to species identification with 16S-PCR and Sanger sequencing. The PCR reaction mix was prepared in a final volume of 25 μL in a thermal cycler (ABI 2720TC, Applied Biosystems, CA, USA): 1× blend master mix buffer, dNTPs (1 mM of each), 0.2 μM forward and reverse primers (P1 and P4 respectively) (Bioneer, Daejeon, Korea), DNA TOP polymerase (1.25 U), DNA (2 μL) and ddH_2_O. PCR reactions consisted οf an initial denaturation step at 94 °C for 5 min, followed by 35 cycles of denaturation at 94 °C, 30 sec, primer annealing at 56 °C, 30 sec and primer extension at 72 °C, 1 min, and concluded by a final extension step at 72 °C for 5 min. After PCR product purification pureLink DNA purification kit (Thermo Scientific, Massachusetts, USA) according to the manufacturer recommendations, cycle sequencing uses the BigDye Terminator v3.1 Sequencing Buffer (Applied Biosystems, CA, USA). The cycle sequencing mix was prepared by mixing the following: 2 μL 2.5X ready reaction premix (ddNTPs), 1 μL 5× BigDye sequencing buffer (polymerase), 1 μL 10 pmol primer, template and 6 μL water. This freshly prepared cycle sequencing reaction mix (9 μL) was added to a reaction tube followed by the addition of 1 μL of the purified PCR products. This tube was placed in a thermal cycler (ABI 2720TC, USA), which was set to perform the following sequencing cycles: initial denaturation step at 96 °C for 1 min, followed by 25 cycles of denaturation at 96 °C, 10 sec, primer annealing at 50 °C, 5 sec and primer extension at 60 °C, 6 min. Afterwards, MagSi-DNA cleanFIX kit reagent (10 μL) (Amsbio, Abingdon, UK), was placed for the removal of unused dNTPs, in the tube consisting of the cycle sequencing product, as described by the manufacturer (Amsbio, Abingdon, UK). 

The nucleotide base sequence of the 16S rRNA gene of the isolated LAB were determined by Sanger sequencing. The Sanger sequencing was carried out by pipetting the final supernatant into a 96-well plate (Applied Biosystems, California, USA). The 3500 Genetic Analyzer (Applied Biosystems, California, USA), was used to perform Sanger sequencing by capillary electrophoresis and analysis involved the inputting of the sequences produced into an NCBI BLAST database.

### 2.5. Statistical Analysis

Data and statistical analysis were performed using GraphPad Prism v8.0.1 (GraphPad, California, USA). TVC and total LAB counts of milk samples collected in summer and winter for all regions were tested for normality using the D′ Agostino and Pearson test and the Shapiro Wilk’s test. Pearson (for the normally distributed) and Spearman (for the non-normally distributed) tests were applied to assess correlation for the TVC and the viable counts of LAB between summer and winter samples. Finally, two-way ANOVA analysis using a mixed model and Sidak’s post-hoc test for multiple comparisons was performed to determine any significant difference between microbial counts of milk samples collected in summer and winter or different geographical locations. 

## 3. Results

The microbiota of raw Maltese cow milk was investigated and the effect of season and region on microbial counts and microbial diversity was assessed for the first time through the current study. A total of 50 raw Maltese cow milk samples was collected, 25 samples in summer and 25 samples in winter (Table 1). For the geographical assessment of microbial counts of raw milk, seven farms were chosen from the Southern region, eight farms from the South-Eastern region, 1 farm from the central region, three farms from the Northern region and six samples from Gozo. In brief, one sample from each farm resident in the specified regions was collected in summer and in winter, to assess the seasonal effect on microbial counts and microbial diversity.

### 3.1. Microbial Population of Raw Maltese Cow Milk

**Figure 1:**
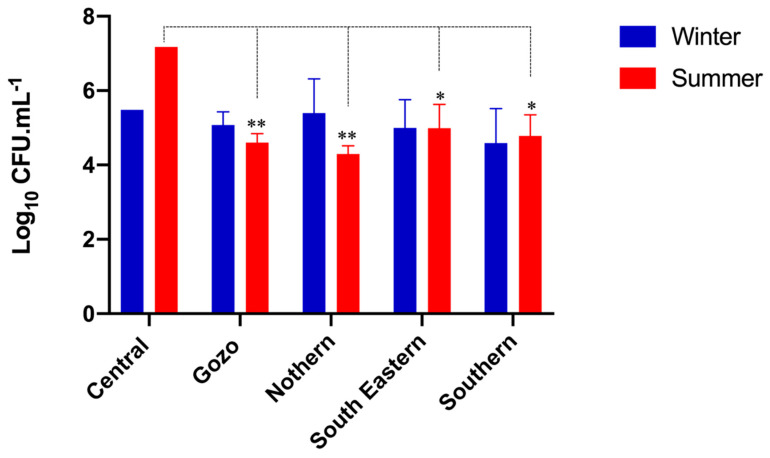
Average total viable counts (TSA counts) presented per geographical region for winter and summer seasons (*p < 0.05, **p < 0.01).

**Figure 2:**
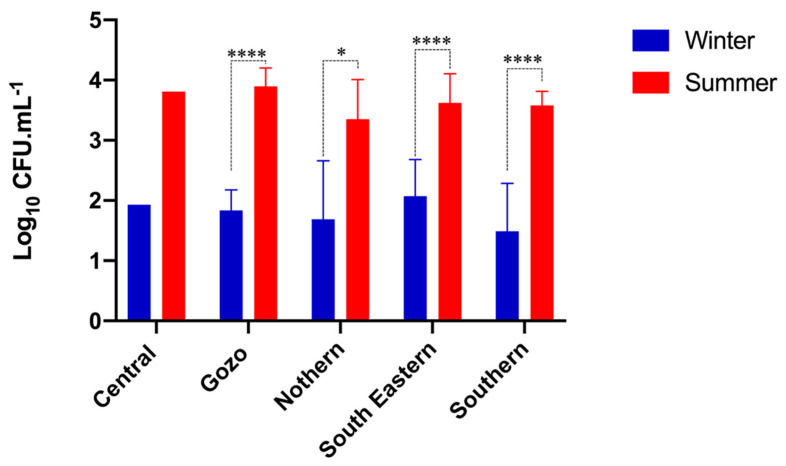
Average lactic acid bacteria counts (LSA counts) presented per geographical region for winter and summer seasons (* *p* < 0.05, **** *p* < 0.0001).

According to the microbial enumeration, TVC in winter and summer samples ranged from 3.22–6.42 log CFU/mL and 3.90–7.17 log CFU/mL, respectively. The LAB population also varied significantly (*p* < 0.0001 based on Two-way ANOVA analysis of pooled geographical samples) between the winter (0.75–3.20 log CFU/mL) and summer samples (2.59–4.33 log CFU/mL) (Table 1). The multiple comparison test indicated that the LAB count was significantly higher for all regions (*p* < 0.05 for Northern and *p* < 0.0001 for Southern, South-Eastern, and Gozo) except the Central region in the summer samples. No correlation between summer and winter samples was found between the TVC (Pearson *r* = 0.2002) and the total LAB counts (Spearman *r* = 0.1423). Analysis for significant differences between different geographical regions results in a significant decrease in TVC when compared to the central region (*p* < 0.05). No significant difference in LAB counts was observed between geographical samples collected in different seasons (Figure 1 and Figure 2). More specifically, in the winter samples, the highest TVC were found in the Northern region, and the lowest in the Southern region. The highest and the lowest LAB population was both enumerated in the Southern region. In the summer samples, the highest TVC were enumerated in the Central region, while the lowest were enumerated in the southern region. The highest LAB population was detected in the South-eastern region, while the lowest were enumerated in the Northern region.

### 3.2. Microbial Diversity of Raw Maltese Cow Milk

**Figure 3:**
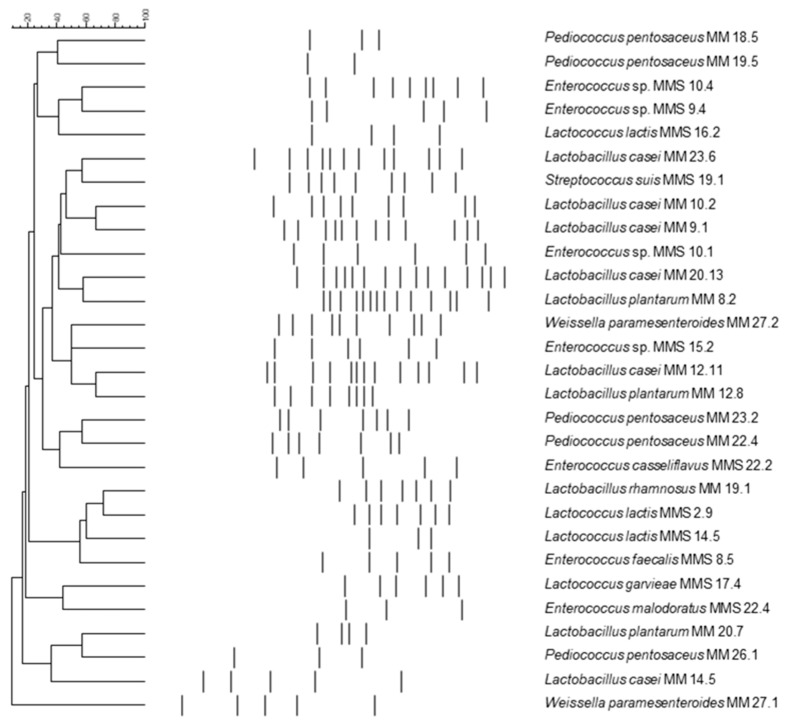
Rep-PCR patterns showing the representative fingerprints of the lactic acid bacteria isolates recovered from different milk samples from Maltese islands. The codes on the right correspond to the strain chosen from either the winter of the summer batch. MM-Maltese milk winter and MMS-Maltese milk summer.

The 262 isolates from raw Maltese cow milk were grouped in 32 clusters based on rep-PCR patterns (Figure 3). The representative isolates from each clusters were assigned to genera *Lactobacillus*, *Pediococcus*, *Weissella*, *Lactococcus*, *Enterococcus*, *Streptococcus*, *Staphylococcus,* and *Bacillus*. In Table 2 the distribution of different clusters in winter and summer samples is shown. It was shown that the majority of the LAB from winter samples were belonging to *Lactobacillus* genus while the majority of the isolates recovered from summer samples were identified as enterococci and *Lactobacillus* spp. 

**Figure 4:**
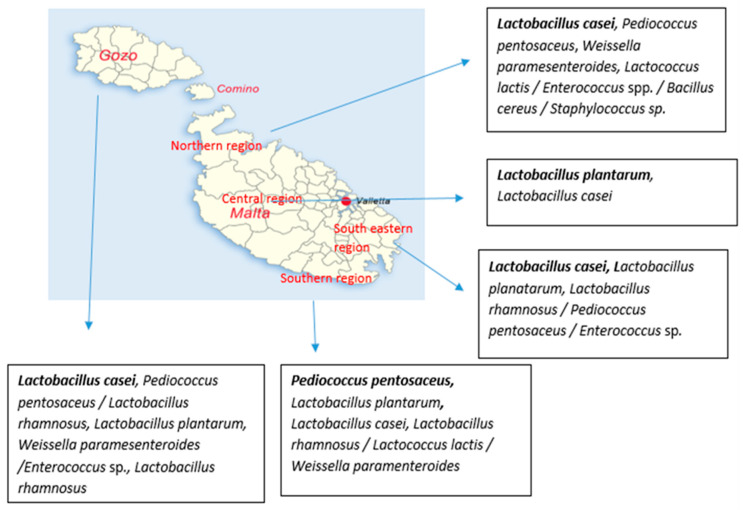
Geographical distribution of isolates at species level from Maltese raw milk recovered from LSA medium during winter period. The species are written from the most commonly detected to fairly detected ones; bold fonts represent the most commonly detected species while the “/” was used to separate the species that were detected in the same number.

The geographical distribution of isolates recovered from LSA medium from raw Maltese milk during winter period at species level is shown in Figure 4. According to the obtained results, *Lactobacillus casei* was the most common recovered species in samples collected from Gozo, Northern and South-eastern region, while *Lactobacillus casei* was also detected in the rest of the regions. Furthermore, *Lactobacillus plantarum* and *Pediococcus pentosaceus* were recovered in higher numbers from samples collected from Central and Southern region, respectively. *Lactobacillus rhamnosus* was fairly recovered from Gozo samples while it was sporadically detected in the south-eastern and southern regions. *Weissella paramesenteroides* was isolated from samples collected from Gozo, Northern and Southern region. It has to be noted that *Bacillus cereus* and *Staphylococcus* sp. were isolated from samples under the Northern region on the geographical distribution map. 

**Figure 5:**
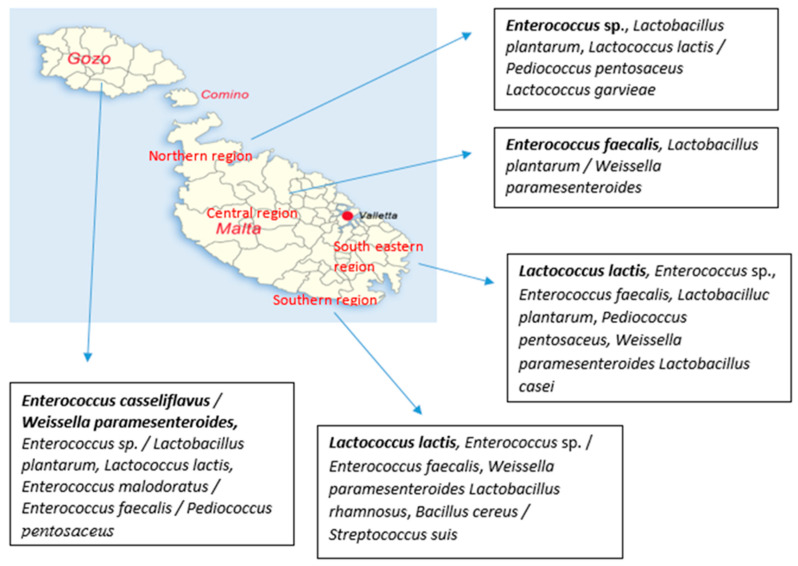
Geographical distribution of isolates at species level from Maltese raw milk recovered from LSA medium during summer period. The species are written from the most commonly detected to rare detected ones; bold fonts represent the most commonly detected species while the “/” was used to separate the species that were detected in the same number.

The geographical distribution of isolates recovered from LSA medium from raw Maltese milk during summer period at species level is shown in Figure 5. In brief, *Enterococcus* was the most common recovered genus in samples collected from Northern (*Enterococcus* sp.) and Central (*Enterococcus faecalis*) region, while its was also detected in high numbers in the rest of the regions. In Gozo samples, *Enterococcus casseliflavus* and *Weissella paramesenteroides* were recovered in higher numbers, with the species *Enterococcus casseliflavus* to be detected only in Gozo samples. However, *Weissella paramesenteroides* was not recovered from Northern region. In the case of *Lactococcus lactis,* the species was recovered in higher numbers from samples collected from South-eastern and Southern region, while it was not detected in Central region. In samples from Southern region, *Lactobacillus plantarum* was not detected, while *Lactobacillus rhamnosus* was recovered only from this region. *Pediococcus pentosaceus* was sporadically detected in Gozo, Northern and South-eastern region. Finally, it has to be noted that *Bacillus cereus* and *Streptococcus suis* were isolated from the Southern region.

Regarding the frequency of isolation, *Weissella paramesenteroides* MM27.2 was the strain that was the most commonly isolated, where a total of 22 similar isolates were recovered from both the summer and winter samples, i.e., 15 from the summer and 7 from the winter samples. In winter, this isolate was found in samples from Gozo, Northern and Southern region, while it was detected in all regions but Central region in summer samples. The next most common isolates were *Enterococcus faecalis* MMS8.5 and *Lactococcus lactis* MMS2.9, where a total of 19 and 16 similar isolates were found respectively, in the summer samples only. *Enterococcus faecalis* was isolated from all regions but Northern region; while *Lactococcus lactis* was isolated from samples of Gozo, South-eastern and Southern region. *Lactobacillus plantarum* MM12.8 and MM8.2, *Lactobacillus casei* (except strain MM10.2) and *Pediococcus pentosaceus* MM19.5 and MM23.2 were also fairly common in the winter isolates *Pediococcus pentosaceus* MM19.5 was only detected in Gozo samples in both periods. Other isolates that were only detected in Gozo samples are *Enterococcus casseliflavus* (summer), *Weissella paramesenteroides* MM27.1 (winter) and *Enterococcus malodoratus* (summer). Similarly, *Lactobacillus casei* MM14.5 was only detected in winter samples of South-eastern region.

## 4. Discussion

### 4.1. Prevalence of Bacteria in Raw Cow’s Milk 

Raw milk was collected from different registered herdsmen from all around Malta and Gozo delivering milk to the local dairy industry. There was quite a different range of bacteria found in each sample indicating that each sample has its own unique bacterial level. The TVC in the milk can indicate the hygienic practices of the farm. In an earlier study, the effect of hygiene practices and disinfection procedures on microbial composition of raw milk was highlighted (Mallet et al. 2012). This is of importance as, an extremely high bacterial count before pasteurization can overwhelm the thermal destruction capacity of the pasteurizer, which can result in pasteurized milk with high bacterial numbers, hence being of inferior quality and which may have a reduced shelf life. Microbial enumeration in this study indicated that some farms have adequate hygienic practice, while other farms require a drastic decrease of the TVC to improve the overall quality of the milk supplied to the local dairy industry. It seems that increased awareness, especially during the summer period, should aim to decrease or prevent contamination of raw milk with bacteria, especially pathogenic bacteria and will result in better quality raw milk supplied to the dairy industry. 

### 4.2. Seasonal and Geographical Prevalence of Bacteria in Raw Cow’s Milk

Statistical analysis performed in this study confirmed that although the summer samples had slightly higher counts of bacteria, there was still no significant difference between the TVC in summer and winter in most raw milk samples. In some cases, the TVC were higher in winter than in summer samples. On the other hand, since only a few differences were noted between summer and winter bacterial counts, these results also indicate that seasonality is not a critical factor for the levels of bacteria in raw milk. No significant difference between summer and winter population of total bacteria in raw milk samples have been also reported earlier [8,9]. However in a recent study, the bacterial counts found to be affected by the sampling month of cow’s milk [10]. In another study, bacterial counts were higher in summer than in winter raw milk samples, indicating that the difference between the total bacterial populations in winter and summer pasteurized milk samples could be due to increased competition within the bacterial community in raw milk during the summer period [11]. 

Although data collected in this study show that the average summer raw milk samples had higher counts of LAB than winter samples, it was also determined that the level at which the bacteria decrease (from summer to winter samples), was different for all the farms. Furthermore, for a small percentage of farms, the winter raw milk samples showed higher counts than summer raw milk samples. In an earlier study, variations in bacterial counts of raw cow’s milk were observed in 10 different farms in South Dakota, which were also related to the sampling season [8]. Similar observations were reported in a previous study, in which the ratio of increased bacterial count varied significantly when comparing summer to winter samples [12]. Salman and Elnasri [13] reported that more LAB were present in summer than in winter samples, where the acidity of the milk was also higher in the summer samples, and the possible reason for this is the higher amount of lactic acid in the milk (produced by the LAB). Since these observations were recorded in various studies, this could indicate that seasonal effect is not the only causative agent leading to an increase in bacterial counts from summer to winter. There might be other farm-specific factors, such as the hygienic practices at the farm, the type food supplied to the herd, and the type of equipment used to milk the herd that can have an effect on the total bacterial population. 

### 4.3. Effect of Seasonality and Geographical Area on Distribution of Different Species Isolated from Raw Cow’s Milk 

It was reported before that microbial enumeration is not enough to highlight the differences in microbial community [14]. This statement is in line with the observations of the present study in which no significant changes of total counts of LAB between the samples of the same region in different sampling season were detected, although different species were identified between the analyzed samples. A wide variety of microorganisms were isolated from raw Maltese milk in this study including: *Lactobacillus, Lactococcus, Bacillus, Enterococcus, Streptococcus,* and also *Staphylococcus* species. In a similar study, a large number of isolates were identified as *Lactococcus lactis* subsp. *lactis*, while *Lactococcus lactis* subsp. *biovar diacetlyactis*, *Lactococcus lactis* subsp. *cremoris*. *Lactoccocus lactis* subsp. *lactis Lactobacillus* and enterococci were found in raw milk in western Algeria [15]. *Lactococcus piscium, Lactococcus lactis, Staphylococcus pasteuri, Staphylococcus warneri,* and *Staphylococcus epidermidis* were detected in raw cow’s milk collected from four farms in Campania [16]. Certain farming practices had an effect on the distribution of species of LAB in the various raw goat’s milk samples from three different regions in France, where *Enterococcus* and *L. lactis* were detected [17]. *Enterococcus* spp., *Lactococcus* spp., *Lactobacillus* spp., *Streptococcus* spp., and *Leuconostoc* spp. were also detected in raw camel milk [18]. In the North-western part of Italy, *Lactobacillus paracasei, Lactococcus lactis, Carnobacterium maltaromaticum, Leuconostoc, Enterococcus* and *Streptococcus* were detected in raw donkey milk [19].

In this study, different genera were the most commonly detected in winter (mainly *Lactobacillus*) and summer (mainly *Enterococcus*) samples. Furthermore, it was observed that some species were only found in the winter sample i.e., *Staphylococcus* sp. or in the summer samples i.e., *Enterococcus casseliflavus, Enterococcus faecalis, Enterococcus malodoratus*, *Streptococcus suis*. Similarly different strains of the same species i.e., *Bacillus cereus* detected only in the winter samples and in summer samples. Furthermore, season seemed to affect the presence of specific strains of the same species, for example in the case of *Lactobacillus casei* five strains were detected only in winter samples while the sixth strain was isolated from both seasons. In another study, where species of the genera *Lactobacillus, Pediococcus* and *Lactococcus* (previously grouped as *Streptococcus*) were isolated from 20 samples of raw Sudanese milk, *Pediococcus* was isolated widely from the winter samples [19]. In recent studies, the microbial composition of cow’s milk found to be also affected by the sampling period [10,20,21]. 

Geographical area seemed to affect also the microbial diversity. More specifically, the most commonly detected species were varied in the samples collected from the different sampling regions. *Lactobacillus casei* was the most common species detected in three regions, while the samples from the other two were dominated by *Lactobacillus plantarum* and *Pediococcus pantosaseus*. On the other hand, enterococci and *Lactococcus lactis* dominated the microbiota of samples from three and two different regions during the summer, respectively. However, the physicochemical composition of Maltese cow milk was not significantly affected when milk from Malta and Gozo farms were compared in two recent studies [6,7]. Similar to our results, Bokulich et al. [22] reported that *Lactobacillus* and *Streptococcus* species were the dominant bacteria isolated from different types of milk in regions throughout Armenia and Georgia followed by *Lactococcus* and *Enterococcus*. The abundance of *Lactococcus* and *Lactobacillus* in Alpine cow milk was found to be higher than in the farm of Trentino [10]. Doyle et al. [23] observed differences in the microbiome of milk collected indoor or outdoor of the same farm.

## 5. Conclusions

Through the present study, the diversity of indigenous lactic acid bacteria in the Maltese cow milk was monitored for the first time. Microbial enumeration of the LAB population resulted in the detection of higher LAB counts in summer when compared to the winter sample. It was also concluded that samples from different herdsmen had different initial TVC and LAB counts. These results enabled the conclusions that in the summer, higher temperatures provide a more adequate environment for LAB to thrive and also, seasonality is not a critical factor determining the total bacterial levels in raw milk. A wide range of bacteria were isolated and identified from the raw cow’s milk. Seasonal differences at total LAB and species/strain level between summer and winter raw milk samples were noted. In total, a variety of different LAB were isolated from the winter and summer samples, where the majority of them were identified as *Lactobacillus* (mainly *Lactobacillus casei* group) and *Enterococcus*, respectively. These results allow us to come to the conclusion that there is a difference between the natural microbiota present in raw Maltese milk during the winter and summer seasons. In conclusion, population dynamics strengthen our knowledge that differences in microbial communities could be observed although similar microbial counts were enumerated. In addition, it was also highlighted that the effect of season and geographical distribution on microbial communities could not be underestimated in microbial studies.

## Figures and Tables

**Table 1:** The total viable counts (TVC) and lactic acid bacteria (LAB) population in both the winter (2015) and summer (2016) raw cow milk samples of Maltese islands.

		Winter Samples		Summer Samples	
Region	Farmer Code	Average TVC (log CFU/mL)	Average Total Counts of LAB (log CFU/mL)	Average TVC (log CFU/mL)	Average Total Counts of LAB (log CFU/mL)
Central	8	5.48 ± 0.10	1.93 ± 0.19	7.17 ± 0.12	3.81 ± 0.03
Gozo	20	5.58 ± 0.07	2.49 ± 0.06	4.79 ± 0.03	4.06 ± 0.01
	21	5.00 ± 0.03	1.71 ± 0.04	4.68 ± 0.02	4.00 ± 0.06
	22	4.69 ± 0.05	1.81 ± 0.02	4.69 ± 0.09	4.13 ± 0.01
	23	4.77 ± 0.12	1.77 ± 0.16	4.13 ± 0.07	3.29 ± 0.06
	24	4.98 ± 0.09	1.69 ± 0.04	4.62 ± 0.07	3.89 ± 0.09
	25	5.42 ± 0.02	1.50 ± 0.05	4.70 ± 0.05	3.99 ± 0.03
Northern	15	6.42 ± 0.02	0.89 ± 0.05	4.08 ± 0.01	2.59 ± 0.11
	16	5.13 ± 0.25	2.77 ± 0.01	4.53 ± 0.01	3.71 ± 0.01
	17	4.63 ± 0.21	1.38 ± 0.01	4.26 ± 0.04	3.75 ± 0.15
South-Eastern	2	5.35 ± 0.05	1.70 ± 0.05	4.89 ± 0.31	4.02 ± 0.02
	3	5.73 ± 0.02	1.90 ± 0.11	5.02 ± 0.24	3.61 ± 0.04
	4	5.26 ± 0.09	2.73 ± 0.19	5.95 ± 0.23	4.33 ± 0.03
	6	4.40 ± 0.02	2.13 ± 0.01	5.12 ± 0.12	3.82 ± 0.02
	7	3.80 ± 0.01	0.88 ± 0.19	5.28 ± 0.01	3.75 ± 0.11
	10	4.34 ± 0.02	2.42 ± 0.21	3.98 ± 0.02	3.06 ± 0.01
	12	6.02 ± 0.04	2.02 ± 0.05	5.42 ± 0.01	3.50 ± 0.07
	14	5.03 ± 0.09	2.75 ± 0.24	4.22 ± 0.04	2.86 ± 0.01
Southern	1	5.25 ± 0.07	1.58 ± 0.05	5.52 ± 0.28	3.82 ± 0.07
	5	4.17 ± 0.08	1.33 ± 0.01	5.28 ± 0.05	3.29 ± 0.02
	9	4.43 ± 0.02	1.13 ± 0.18	4.70 ± 0.04	3.87 ± 0.01
	11	4.37 ± 0.01	1.27 ± 0.04	5.14 ± 0.02	3.29 ± 0.17
	13	3.21 ± 0.17	0.75 ± 0.15	3.90 ± 0.02	3.61 ± 0.01
	18	4.43 ± 0.49	1.13 ± 0.83	4.33 ± 0.07	3.46 ± 0.03
	19	6.21 ± 0.12	3.19 ± 0.14	4.56 ± 0.11	3.69 ± 0.08

**Table 2:** Identified strains by sequencing and the number of isolates that similar patterns were obtained by rep-PCR from the total amount of isolates recovered from the winter and summer raw cow milk samples of Maltese islands.

Group	Species	Strain	Similar Pattern	Winter	Summer
I	*Enterococcus* sp.	MMS 10.4	11	2	9
II	*Enterococcus casseliflavus*	MMS 22.2	7	0	7
III	*Enterococcus faecalis*	MMS 8.5	19	0	19
IV	*Lactococcus lactis*	MMS 2.9	16	0	16
V	*Lactobacillus rhamnosus*	MM 19.1	14	12	2
VI	*Lactococcus garvieae*	MMS 17.4	2	1	1
VII	*Lactobacillus plantarum*	MM 12.8	11	11	0
VIII	*Enterococcus* sp.	MMS 15.2	7	5	2
IX	*Lactobacillus plantarum*	MM 8.2	12	12	0
X	*Lactobacillus casei*	MM 12.11	12	12	0
XI	*Lactobacillus casei*	MM 9.1	10	10	0
XII	*Lactobacillus casei*	MM 20.13	8	8	0
XIII	*Lactobacillus casei*	MM 23.6	11	11	0
XIV	*Weissella paramesenteroides*	MM 27.2	22	7	15
XV	*Pediococcus pentosaceus*	MM 23.2	10	10	0
XVI	*Pediococcus pentosaceus*	MM 22.4	8	4	4
XVII	*Enterococcus* sp.	MMS 10.1	7	0	7
XVIII	*Lactobacillus casei*	MM 10.2	4	2	2
XIX	*Lactobacillus plantarum*	MM 20.7	17	3	14
XX	*Enterococcus* sp.	MMS 9.4	6	0	6
XXI	*Lactococcus lactis*	MMS 16.2	3	1	2
XXII	*Lactococcus lactis*	MMS 14.5	16	1	15
XXIII	*Pediococcus pentosaceus*	MM 18.5	3	3	0
XXIV	*Pediococcus pentosaceus*	MM 26.1	4	2	2
XXV	*Pediococcus pentosaceus*	MM 19.5	10	8	2
XXVI	*Lactobacillus casei*	MM 14.5	4	4	0
XXVII	*Weissella paramesenteroides*	MM 27.1	2	2	0
XXVIII	*Enterococcus malodoratus*	MMS 22.4	2	0	2
XXIX	*Streptococcus suis*	MMS 19.1	1	0	1
XXX	*Bacillus cereus*	MMS 1.4	1	0	1
XXXI	*Staphylococcus* sp.	MM 16.9	1	1	0
XXXII	*Bacillus cereus*	MM 16.2	1	1	0
